# Praf2 Is a Novel Bcl-xL/Bcl-2 Interacting Protein with the Ability to Modulate Survival of Cancer Cells

**DOI:** 10.1371/journal.pone.0015636

**Published:** 2010-12-20

**Authors:** Maria Teresa Vento, Valeria Zazzu, Alessia Loffreda, Justin R. Cross, Julian Downward, Maria Patrizia Stoppelli, Ingram Iaccarino

**Affiliations:** 1 Institute of Genetics and Biophysics “Adriano Buzzati-Traverso,” Consiglio Nazionale delle Ricerche (CNR), Naples, Italy; 2 Signal Transduction Laboratory, Cancer Research UK, London Research Institute, London, United Kingdom; Universidade Federal do Rio de Janeiro, Brazil

## Abstract

Increased expression of Bcl-xL in cancer has been shown to confer resistance to a broad range of apoptotic stimuli and to modulate a number of other aspects of cellular physiology, including energy metabolism, cell cycle, autophagy, mitochondrial fission/fusion and cellular adhesion. However, only few of these activities have a mechanistic explanation. Here we used Tandem Affinity purification to identify novel Bcl-xL interacting proteins that could explain the pleiotropic effects of Bcl-xL overexpression. Among the several proteins co-purifying with Bcl-xL, we focused on Praf2, a protein with a predicted role in trafficking. The interaction of Praf2 with Bcl-xL was found to be dependent on the transmembrane domain of Bcl-xL. We found that Bcl-2 also interacts with Praf2 and that Bcl-xL and Bcl-2 can interact also with Arl6IP5, an homologue of Praf2. Interestingly, overexpression of Praf2 results in the translocation of Bax to mitochondria and the induction of apoptotic cell death. Praf2 dependent cell death is prevented by the co-transfection of Bcl-xL but not by its transmembrane domain deleted mutant. Accordingly, knock-down of Praf2 increases clonogenicity of U2OS cells following etoposide treatment by reducing cell death. In conclusion a screen for Bcl-xL-interacting membrane proteins let us identify a novel proapoptotic protein whose activity is strongly counteracted exclusively by membrane targeted Bcl-xL.

## Introduction

The acquired capability to escape apoptosis is required at several steps during cancer development. Over-expression of the Bcl-xL protein is known to confer resistance to a broad range of potentially apoptotic stimuli arising during cancer development, such as oncogene activation, hypoxia and matrix detachment [1]–[3]. Impaired apoptosis due to the over-expression of the Bcl-xL gene is therefore critical during cancer progression. Impaired apoptosis is also a major barrier to effective cancer treatment, because cytotoxic therapies for cancer strongly rely on induction of apoptosis. Interestingly, Bcl-xL has been suggested to play a unique role in general resistance to cytotoxic agents, because of a striking correlation between an increased Bcl-xL expression level and resistance to a wide panel of standard chemotherapy agents. Bcl-xL's mechanism of action is therefore a major component of chemoresistance in cancer cells [4]. Bcl-xL belongs to the Bcl-2 family of proteins whose members can have either anti-apoptotic or pro-apoptotic functions. The proapoptotic members of the Bcl-2 family fall into two subsets. The so-called multidomain factors (Bax and Bak) are proteins sharing more than one Bcl-2 Homology (BH) domain. The other subfamily comprises proteins sharing only the BH3 domain. A picture has emerged suggesting that “BH3 only” proteins have diverse mechanisms of regulation and are targeted sensors of different sources of cell stress. Their primary function appears to be the binding and neutralization of the anti-apoptotic Bcl-2 family membres [5], although some of them have also been reported to be able to directly activate multidomain proapoptotic family members [6], [7]. It is therefore widely accepted that elevated Bcl-xL protein level decreases susceptibility to apoptosis because it increases the cellular potential to inactivate pro-apoptotic “BH3 only” proteins [8].

Besides its ability to inhibit the core apoptotic machinery, Bcl-xL has been shown to modulate a number of other aspects of cellular physiology. Its overexpression, for instance, has been correlated with high tumour grade and increased ability to invade and metastasize, independently of its ability to sustain survival in the absence of matrix attachment [9]–[11]. Bcl-xL has been found to complement *S.cerevisiae* genes that facilitate the switch from glycolytic to oxidative metabolism [12]. Bcl-xL is also able to modulate calcium homeostasis [13], stimulate synapse formation [14], slow cell cycle progression [15], modulate autophagy [16], [17], increase mitochondrial fission/fusion [18] and modulate metabolite exchange across the outer mitochondrial membrane [19]. Some of these “unconventional” Bcl-xL activities could be explained by its ability to interact with proteins other than the pro-apoptotic “BH3 only” factors. Bcl-xL has indeed been shown to interact with VDAC1 [20], with the IP3 Receptor [21], with Beclin1 [22] and a number of other proteins.

Bcl-xL is both a cytosolic and a membrane-associated protein [23]. While cytosolic Bcl-xL appears to be a homodimer [24], the quaternary structure of membrane-bound Bcl-xL has not been investigated in details, although it has been reported that it could be engaged in high molecular weight complexes [25] (Borner personal communication). In the present study we present evidence that Bcl-xL is indeed part of high molecular weight complexes and we attempt to carry a comprehensive analysis of proteins able to bind Bcl-xL using Tandem Affinity Purification. Interestingly, we found that Bcl-xL interacts with proteins involved in several cellular processes. Among them, the secretory pathway was one of the most represented pathways. With the aim of validating the list of proteins found to interact with Bcl-xL, we decided to focus our analysis on the interaction between Bcl-xL and Praf2, a small protein belonging to the Prenylated Rab Acceptor family, with a predicted role in ER to Golgi transport [26]. We show that Praf2 induces apoptotic cell death upon expression and that Bcl-xL counteracts Praf2's apoptotic activity. Finally, we show that Praf2 silencing in U2OS cells makes them more resistant to apoptosis induced by the cytotoxic drug etoposide.

## Results

**Figure 1:**
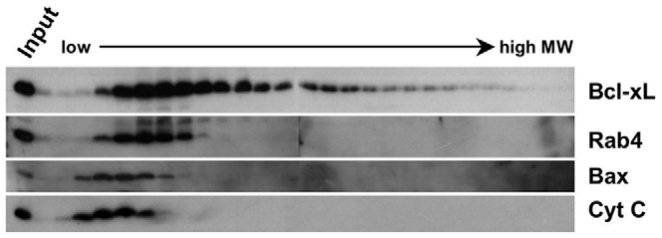
Bcl-xL is present in a wide range of molecular weights in fractionated U2OS cell extracts. Sucrose gradient fractionation of U2OS total cell extracts. U2OS (60×10^6^ cells) were lysed in CHAPS extraction buffer and cleared by centrifugation. Extracts were fractionated using a 10–40% sucrose gradient and equal volumes of the fractions were analysed by immunoblot for the presence of Bcl-xL, Bax, Rab4 and Cytochrome c.

To investigate the possibility that Bcl-xL is associated with membrane proteins in high molecular weight complexes, we have used sucrose gradient fractionations of CHAPS-solubilized whole cells extracts. The analysis was performed using the U2OS cell line because it expresses high levels of Bcl-xL and appears to be “addicted” to Bcl-xL expression for survival (not shown). Pre-cleared cell extracts were loaded on sucrose gradients and subjected to ultracentrifugation as specified in Materials and Methods. Fractions were collected, resolved by SDS-PAGE and analysed by immunoblot. Figure 1 shows that low molecular weight proteins like Cytochrome c (12 kDa), Rab 4 (25 kDa) or Bax (20 kDa) are present in fractions of the gradient where low-molecular weight proteins are expected to sediment. Bcl-xL, instead, is spread in fractions ranging from low to high molecular weights. This result suggests that under these experimental conditions Bcl-xL could be engaged in a number of different molecular complexes.

### Establishment of HeLa cells expressing a TAP-Bcl-xL chimera protein

**Figure 2:**
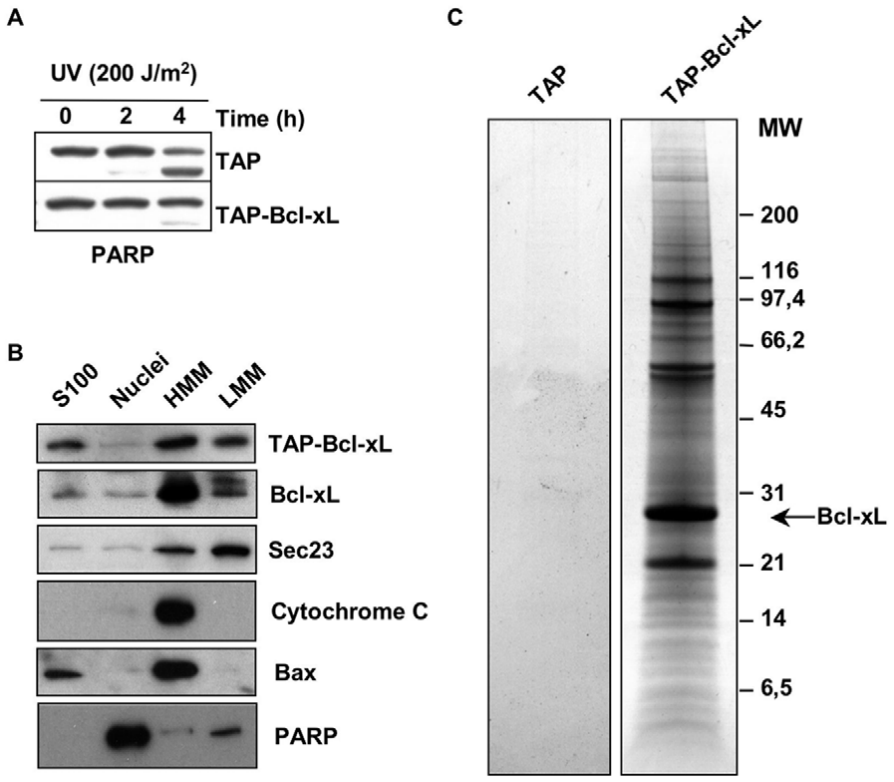
TAP-Bcl-xL is able to protect from apoptosis, localise correctly and co-purify with several cellular proteins. (**A**) HeLa cells stably expressing TAP-tagged Bcl-xL are more resistant than control to UV-induced apoptosis. Analysis of PARP cleavage in HeLa cells expressing TAP alone or TAP-Bcl-xL and irradiated or not with UV (200 J/m^2^). Cells were collected 2 and 4 hours after irradiation and analysed by immunoblot for the appearance of the cleaved product of PARP. (**B**) TAP-tagged Bcl-xL localises to the same intracellular compartment of endogenous Bcl-xL. HeLa cells expressing TAP-Bcl-xL were fractionated by differential centrifugation to obtain: lane 1, cytosolic proteins (S100); lane 2, nuclear proteins (Nuclei); lane 3, the heavy membrane fraction (HMM); lane 4, the light membrane fraction (LMM). 20 µg of each fraction were analysed by immunoblot for the presence of TAP-BclxL (using secondary antibody only) and endogenous Bcl-xL. The quality of the fractionation was determined using the antibodies against Sec23 (LMM), Cytochrome c (HMM), Bax (S100 and HMM) and PARP (Nuclei). (**C**) HeLa cells stably transfected with TAP alone or TAP-Bcl-xL were subjected to Tandem Affinity Purification. The figure shows a representative Coomassie stained SDS-PAGE performed under reducing conditions used to resolve eluates from purifications. Purifications were performed using membrane CHAPS extracts from HeLa expressing TAP alone (lane 1) or HeLa expressing TAP-Bcl-xL (lane 2).

In order to identify the set of proteins interacting with Bcl-xL in the conditions analysed, we made use of Tandem Affinity Purification [27], [28]. Bcl-xL is a tail-anchored protein that acquires membrane localisation inserting its C-terminal hydrophobic tail into the lipid bilayer of several membrane compartments. To avoid interference with Bcl-xL membrane insertion, we generated an expressing vector placing a TAP tag at the N-terminus of Bcl-xL. We also obtained a control vector where a Stop codon was added downstream of the TAP sequence. Both constructs were transfected in HeLa cells to obtain clones stably expressing either TAP-Bcl-xL or TAP-stop only. We chose HeLa cells because they can easily be adapted to growth in suspension cultures, in order to produce starting material suitable for biochemical purifications. Unlike U2OS, HeLa cells, furthermore, have a relatively low expression level of endogenous Bcl-xL (Figure S1), which may compete with TAP-Bcl-xL for binding partners. To test if the addition of the TAP tag to Bcl-xL could impair its ability to protect from apoptosis, we challenged cells expressing TAP-Bcl-xL (HeLa/TAP-Bcl-xL) or TAP-stop (HeLa/TAP) with UV irradiation and analysed the cleavage status of PARP, as a measure of cellular caspase 3 activity. As shown in Figure 2A, compared to TAP-stop, HeLa expressing TAP-Bcl-xL displayed a reduced level of the PARP cleavage product. Therefore, addition of the TAP tag to the N-terminus of Bcl-xL does not prevent the ability of Bcl-xL to protect cells from UV-induced apoptosis. We next tested if the addition of the TAP tag to Bcl-xL could alter its subcellular distribution. HeLa/TAP-Bcl-xL cells were fractionated in cytosolic (S100), nuclear (Nuclei), heavy membranes (HMM) and light membranes (LMM) fractions by differential centrifugation. Subcellular localisation of TAP-Bcl-xL compared to endogenous Bcl-xL was analysed by immunoblot. The analysis has been performed using equivalent amounts of proteins for each given fraction and it is not informative on relative distribution of the proteins analysed in the different cellular compartments. The quality of the fractionation was defined using antibodies against known markers of subcellular compartments: PARP for the nuclear fraction, Sec23 for the LMM fraction (microsomes) and Cytochrome c for the HMM fraction (mitochondria). We also analysed the localisation of Bax, another member of the Bcl-xL protein family, that is known to localise both at the mitochondria an in the cytosol. Figure 2B shows that, similarly to endogenous Bcl-xL, TAP-Bcl-xL localises to the HMM fraction, to the LMM and to the S100 fractions. Therefore, addition of the TAP tag does not interfere with the ability of Bcl-xL to acquire membrane localisation.

### Identification of novel Bcl-xL-interacting proteins using Tandem Affinity Purification

In order to produce a suitable starting material for TAP purifications, HeLa/TAP-Bcl-xL and HeLa/TAP cells were adapted to growth in suspension culture using spinning glass bottles. Given our interest in membrane based interactions we chose to focus on purifications performed using membrane extracts. Cell pellets were mechanically lysed using a Dounce homogeniser in a detergent-free hypotonic buffer. Centrifugation of the latter cell lysate allowed us to separate total membranes from cytosolic proteins. Total membranes were extracted using a CHAPS containing buffer, and subjected to tandem affinity purification. Eluates from the last purification step were concentrated and resolved on SDS-PAGE. Fig. 2C shows representative pictures of TAP purifications analysed on Coomassie stained gels from TAP-only expressing cells (lane 1) or TAP-Bcl-xL expressing cells (lane 2). Little material arose from the purifications made from TAP only expressing cells, indicating that the background given by unspecific adsorption to the chromatographic media used is negligible. Bands corresponding to the most abundant protein species were cut from the gels and their identity was assessed by LC-MS/MS. A list of protein found to co-elute with TAP-Bcl-xL is shown in Table 1.

**Table 1:** Bcl-xL interacts with proteins involved in several cellular processes.

Name	Description	Gene Bank	UniProt	Loc	Known
*Bcl-2 family members*					
Bad	BCL2-associated agonist of cell death	NP_004313	Q92934	Cyto/Mito	✓
Bax	BCL2-associated X protein	NP_004315	Q07812	Cyto/Mito/ER	✓
Bak	BCL2-antagonist/killer 1	NP_001179	Q16611	Mito/ER	✓
Bid	BH3 interacting domain death agonist	NP_932070	P55957	Cyto/Mito	✓
BBC3	BCL2 binding component 3	NP_001120713	Q9BXH1	Cyto/Mito	✓
Bim	BCL2-like 11 (apoptosis facilitator)	NP_619527	O43521	Cyto/Mito	✓
*Mitochondrial energy metabolism and physiology*					
ATP5A1	ATP synthase alpha subunit	NP_004037	P25705	Mito	
ATP5B	ATP synthase beta subunit	NP_001677	P06576	Mito	
ATP5O	ATP synthase OSCP subunit	NP_001688	P48047	Mito	
ATP5D	ATP synthase delta subunit	NP_001678	P30049	Mito	
ATP5F1	ATP synthase b subunit	NP_001679	P24539	Mito	
ATP5J2	ATP synthase f subunit	NP_001003713	P56134	Mito	
ATP5L	ATP synthase g subunit	NP_006467	O75964	Mito	
ATP5I	ATP synthase e subunit	NP_009031	P56385	Mito	
ATP5H	ATP synthase d subunit	NP_001003785	O75947	Mito	
ATP5C1	ATP synthase gamma chain	NP_001001973	P36542	Mito	
MT-CO2	Cytochrome c oxidase Subunit II	NP_536846	P00403	Mito	
COX4I1	Cytochrome c oxidase Subunit IV	NP_001852	P13073	Mito	
CYB5B	Cytochrome b5 type B	NP_085056	O43169	Mito	
CPS1	Carbamoyl phosphate synthetase 1	NP_001116105	P31327	Mito	
VDAC1	Voltage-dependent anion channel 1	NP_003365	P21796	Mito	✓
VDAC2	Voltage-dependent anion channel 2	NP_003366	P45880	Mito	
PHB2	Prohibitin 2	NP_001138303	Q99623	Mito	
STOML2	stomatin (EPB72)-like 2	NP_038470	Q9UJZ1	Mito	
*Transporters*					
SLC3A2	Solute carrier family 3, member 2	NP_001012679	P08195	MEM	
ATP1A1	ATPase, Na+/K+ transporting, alpha 1 subunit	NP_000692	P05023	MEM	
ATP2A2	ATPase, Ca++ transporting, SERCA2	NP_733765	P16615	ER	✓
TFRC	Transferrin receptor	NP_001121620	P02786	MEM	
*Trafficking*					
SEC61B	SEC61 beta subunit	NP_006799	P60468	ER	
SEC11A	SEC11 homolog A	NP_776890	P67810	ER	
CANX	Calnexin	NP_001737	P27824	ER	
MPDU1	Manose-P-dolichol utilization defect 1	NP_004861	O75352	ER	
RPN1	Ribophorin I	NP_002941	P04843	ER	
DDOST	dolichyl-diphosphooligosaccharide-protein glycosyltransferase	NP_005207	P39656.3	ER	
SEC22B	Vesicle trafficking protein sec22b	NP_004883	O75396	ER	
TMED10	transmembrane emp24-like protein 10	NP_006818	P49755	Golgi	
ERGIC1	ER-Golgi intermediate compartment 1	NP_001026881	Q969X5	Golgi/ER	
TMED5	Transmembrane emp24 protein transport domain containing 5	NP_057124	Q9Y3A6	Golgi/ER	
Praf2	PRA1 domain family, member 2	NP_009144	O60831	ER	
KDELR1	KDEL endoplasmic reticulum protein retention receptor 1	NP_006792	P24390	ER	
SSR4	Signal sequence receptor delta	NP_006271	P51571	ER	
RAB7A	RAB7A, member RAS oncogene family	NP_004628	P51149	Endo	
VAMP3	Vesicle associated membrane protein 3	NP_004772	Q6FGG2	Endo	
*Unknown*					
IFITM1	Interferon induced transmembrane protein 1	NP_003632	P13164	MEM	
C8orf55	Mesenchymal stem cell protein DSCD75	NP_057731	Q8WUY1	Sec	
FAM162A	E2-induced gene 5 protein	NP_055182	Q96A26	Mito	
SFXN1	Sideroflexin 1	NP_073591	Q9H9B4	Mito	
TMEM109	Transmembrane protein 109	NP_076997	Q9BVC6	ER	
UPF0569	Hypothetical protein LOC203547	NP_001017980	Q3ZAQ7	MEM	
USMG5	Upregulated during skeletal muscle growth 5	NP_116136	Q96IX5	Mito	
CISD2	CDGSH iron sulfur domain 2	NP_001008389	Q8N5K1	ER	

LC-MS/MS identified proteins from TAP purifications performed on total membranes of TAP-Bcl-xL expressing HeLa cells were divided into the functional categories described. The table provides name, description and accession numbers of the identified proteins. The table also states the subcellular localisation (known or putative) of the proteins (Loc) and if the protein had already been found interacting with Bcl-xL (known). Mito: mitochondrial; ER: endoplasmic reticulum; MEM: plasma membrane; Cyto: cytosolic; Sec: secreted.

### Bcl-xL is able to interact with proteins involved in several cellular processes

Although the list of Bcl-xL interacting proteins presented in Table 1 cannot be taken as exhaustive, it can be considered a high confidence list. We have subdivided all identified proteins in different functional categories and for each protein we have specified the known or putative sub-cellular localisation. We have also stated if the protein had already been previously found to interact with Bcl-xL. It is interesting to note that most of anti-apoptotic members of the Bcl-2 protein family that have been previously described to interact with Bcl-xL are present in the final eluate. VDAC1 was also already described to interact with Bcl-xL [20], while VDAC2, a less abundant isoform, has been shown to interact with the multidomain Bcl-2 family member Bak [29]. ATP2A2/Serca2 instead, has been reported to interact with Bcl-2 [30]. Overall the presence of these proteins in the final eluate of our purification constitutes a good indication of the specificity of the interactions found. Besides the Bcl-2 family members, we put together a group of proteins that are all mitochondrial and are functionally involved in energy metabolism and mitochondrial physiology. This group includes 10 of the 17 subunits of the ATP Synthase multiprotein complex, subunits II and IV of the cytochrome c oxidase and cytochrome b5 type B, all part of the biochemical chain responsible for oxidative phosphorylation and ATP production. It also includes mitochondrial carbamoyl-phosphate synthetase 1, which plays an important role in removing excess ammonia from the cell, and VDAC1 and 2, main regulators of mitochondrial permeability. In this group we included two more mitochondrial proteins identified as Bcl-xL associated: Stomatin-like protein 2 (StomL2) and Prohibitin 2. StomL2 has been recently shown to be part of a complex with Mitofusin 2 [31] while Prohibitin 2 is supposed to play a role in the regulation of mitochondrial respiration.

Another set of proteins could be grouped in the functional category of transporters. They are located at different membrane compartments, and are: the amino acid transporter SLC3A2; the cell membrane Na+/K+ transporter ATPase ATP1A1; the ER resident calcium transporter ATPase ATP2A2/Serca2; the transferrin receptor, that could be seen as an atypical transporter. A fourth functional group of proteins directly or indirectly associated with Bcl-xL is composed of proteins with a putative or established role in the secretory branch of intracellular trafficking. Those are components of the translocation complex (Sec61 beta, Sec11A), chaperones involved in protein folding assistance in the ER (Calnexin), proteins involved in ER specific protein modifications (Ribophorin I, OST48, MPDU1) and proteins with a putative role in ER to Golgi trasport (Sec22b, ERGIC-32, TMP21, TMED5, Praf2) as well as ER retention (SSR4, KDEL receptor 1). Other trafficking proteins associated with Bcl-xL were Rab7, a small GTPase with a role in late endosome maturation and VAMP3, a protein with a putative role in endosomes recycling. Finally we identified as novel Bc-xL interacting proteins a group of factors either with unknown or very poorly described function (Table 1).

### Validation of the interaction between Praf2 and Bcl-xL

**Figure 3:**
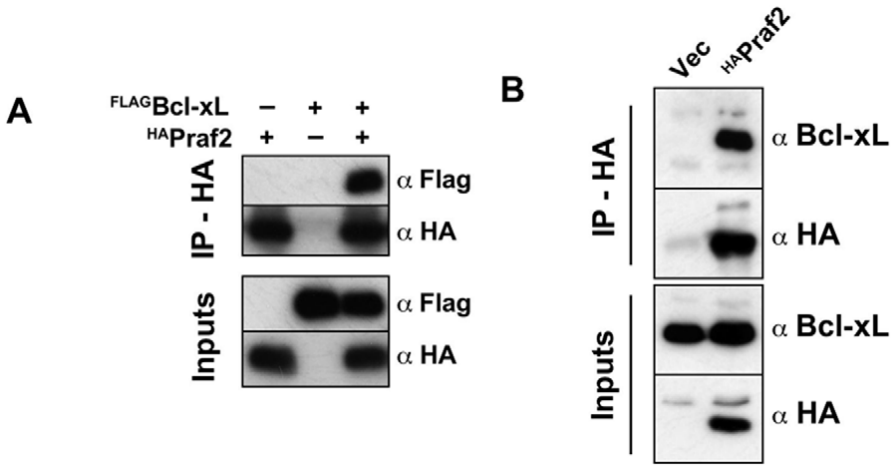
Bcl-xL and Praf2 interact by co-immunoprecipitation. (**A**) HEK 293T cells were either transfected with HA-tagged Praf2 or FLAG-tagged BclxL or co-transfected with both expression vectors. Samples were subjected to immunoprecipitation, using anti HA-conjugated agarose and both total lysates (inputs) and agarose beads eluates (IP) were analysed by immunoblot using anti HA and anti FLAG monoclonal antibodies. (**B**) U2OS cells were transfected with HA-tagged Praf2 or the empty vector (Vec) and cell lysates were subjected to immunoprecipitation, using HA-conjugated agarose. Both lysates and agarose beads were analysed by immunoblot using anti HA and anti Bcl-xL antibodies.

The relationship between the secretory branch of intracellular trafficking and apoptotic cell death is far from being understood. Among the newly identified proteins, Praf2 has been suggested to play a role in ER to Golgi transport [26]. Praf2 belongs to the Prenylated Rab Acceptor (PRA) family of proteins, whose founder, Pra1/Rabac1, has been shown to interact with the viral Bcl-2 homologue BHRF1, and modulate its activity [32]. To gain insights into the relationship between vesicular transport and apoptosis, we decided to focus our attention on Praf2. The cDNA coding for human Praf2 was obtained from the IMAGE consortium and cloned in pcDNA3 with a triple HA tag fused at its C-terminus. In order to validate the interaction between Praf2 and Bcl-xL, 293T cells were transfected either with Praf2^HA^ and ^FLAG^Bcl-xL expression plasmids alone or co-transfected with both plasmids. 24 hours after transfections cells were lysed and Praf2 was immunoprecipitated using monoclonal anti-HA conjugated agarose. As shown in Fig. 3A, ^FLAG^Bcl-xL was detectable in the precipitate only when co-expressed with Praf2. We were therefore able to confirm the interaction of Bcl-xL and Praf2 also in a two-components system, where Bcl-xL and Praf2 are concomitantly overexpressed.

The finding that Praf2 co-purified with Bcl-xL by Tandem Affinity Purification clearly demonstrates that Bcl-xL is able to interact with endogenous Praf2 in HeLa cells. We now investigated if endogenous Bcl-xL is able to interact with Praf2 in U2OS cells. U2OS cells were transfected either with empty vector or with the Praf2^HA^ expressing vector, and lysates were immunoprecipitaed using anti HA-conjugated agarose. As shown in Fig. 3B, anti HA-conjugated agarose can specifically precipitate endogenous Bcl-xL in U2OS cells transfected with Praf2^HA^ but not with the empty vector.

### Multiple members of the Bcl-2 family are able to interact with multiple members of the PRA family

**Figure 4:**
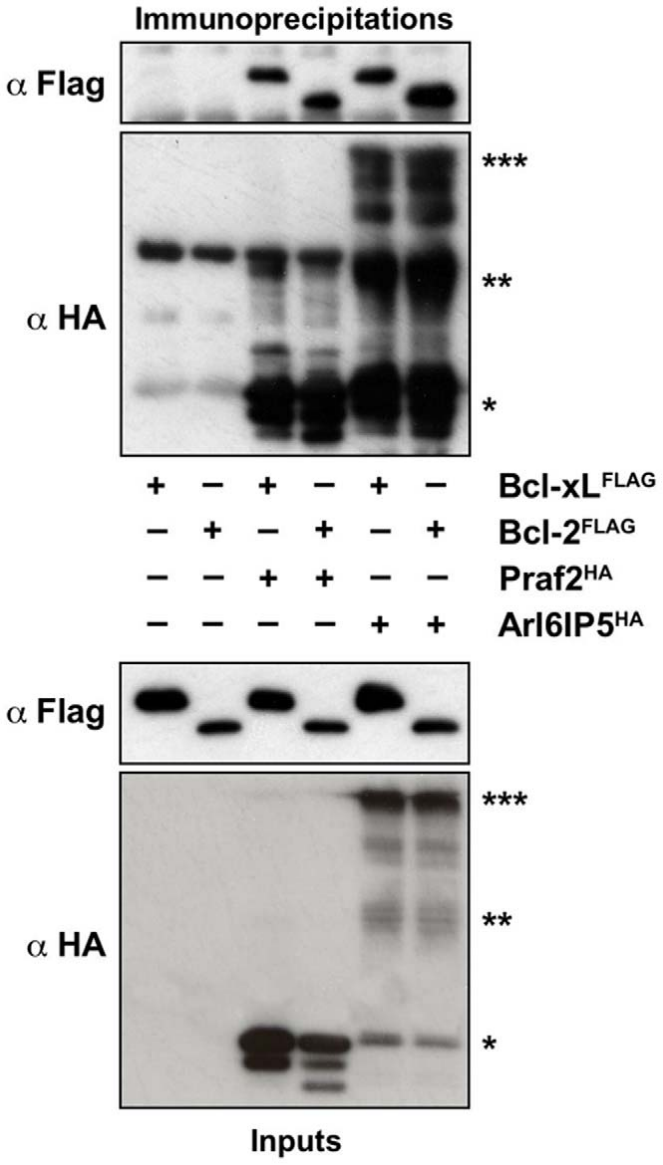
Praf2 and Arl6IP5 interact also with Bcl-2. HEK 293 cells were either transfected with FLAG-tagged BclxL or Bcl-2 alone (lane 1 and 2), or co-transfected with HA-tagged Praf2 or HA-tagged Arl6IP5 (lanes 3 to 6). Samples were subjected to immunoprecipitation, using HA-conjugated agarose and both lysates and agarose beads were analysed by immunoblot using anti HA and anti FLAG antibodies. Arl6IP5 monomer: *; Arl6IP5 dimer: **; Arl6IP5 multimer: ***.

The founder member of the PRA family of proteins, Pra1/Rabac1, has been shown to interact with the viral Bcl-2 homologue BHRF1, but not with Bcl-2 itself [32]. We therefore asked if the binding ability of Praf2 was limited to Bcl-xL or could be extended to Bcl-2 as well. We also asked if the close homologue of Praf2, Arl6IP5, is also able to interact with Bcl-xL and/or Bcl-2. Fig. 4 shows that Praf2^HA^ can interact also with ^FLAG^Bcl-2 (lane 4), and also that, Arl6IP5^HA^ can interact with both ^FLAG^Bcl-xL and ^FLAG^Bcl-2 (lane 5 and 6). Arl6IP5^HA^ expression results in bands with different electrophoretic mobility. Given that they are all immunoprecipitated by the anti-HA antibody and given that it has been described that both Praf2 and Arl6IP5 can give rise to SDS-insoluble species [33], we believe the bands observed correspond to monomers, dimers and multimers of Arl6IP5.

### The transmembrane domain of Bcl-xL is essential for its interaction with Praf2

**Figure 5:**
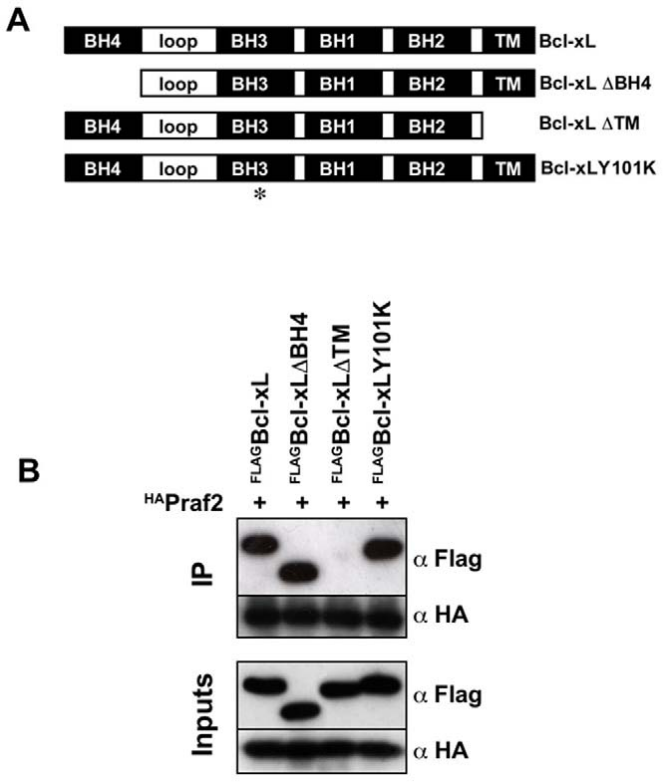
Deletion of Bcl-xL's transmembrane domain prevents its interaction with Praf2. (**A**) Schematic representation of the Bcl-xL expression constructs used in this study. Bcl-xL: full length Bcl-xL; Bcl-xLΔBH4: Bcl-xL lacking the first 24 aminoacids; Bcl-xLΔTM: Bcl-xL lacking the last 22 aminoacids; Bcl-xLY101K: Bcl-xL with a substitution of Tyrosine 101 with Lysine. (B) HEK 293 cells were co-transfected with HA-tagged Praf2 and the BclxL constructs described in (A) carrying a FLAG-tag at the N-terminus. Samples were subjected to immunoprecipitation, using HA-conjugated agarose and both lysates and agarose beads were analysed by immunoblot using anti HA and anti FLAG antibodies.

The ability of Bcl-xL to interact with the pro-apoptotic members of the Bcl-2 family is known to be mediated by a hydrophobic cleft formed on its surface by the BH1, BH2 and BH3 domains [34]. Conversely, the binding of Bcl-2/xL to Raf and Ras has been shown to depend on the BH4 domain [35], [36]. In order to define the domains of Bcl-xL responsible for its interaction with Praf2, we generated a Bcl-xL mutant deleted of its first 24 aminoacids corresponding to the BH4 domain (Bcl-xLΔBH4). We also generated a Bcl-xL mutant in which tyrosine 101 was replaced with a lysine (Bcl-xLY101K) (Fig. 5A), a mutation that has been described to abolish Bcl-xL's ability to interact with Bax [37]. Finally, because Praf2 is a membrane protein [33], we generated a Bcl-xL mutant deleted of its C-terminal 22 aminoacids corresponding to its transmembrane domain (Bcl-xLΔTM). As shown in Fig. 5B, neither deletion of the BH4 domain nor the Y101K mutation affected Bcl-xL's ability to interact with Praf2. However, deletion of the C-terminal transmembrane domain, completely abolished Praf2/Bcl-xL interaction, indicating that the TM domain is essential to this interaction.

### Praf2 overexpression induces apoptotic cell death

**Figure 6:**
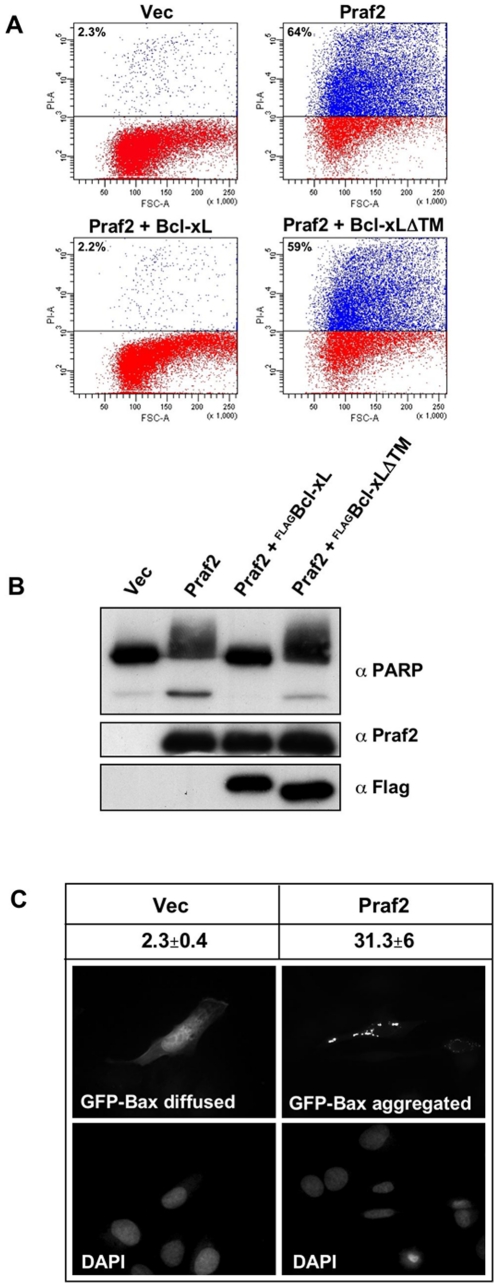
Expression of Praf2 induces apoptotic cell death. (**A**) FACS analysis of HeLa cells transfected with the indicated expression vectors and stained with Propidium Iodide (PI). Percentages of PI positive cells are indicated on the top-left of each graph. (**B**) Aliquots of the HeLa cells transfected as in (A) were analysed by immunoblot for the appearance of the cleaved form of PARP. Expression levels of Praf2, Bcl-xL and Bcl-xLΔTM are also indicated. (**C**) Analysis of GFP-Bax localisation in U2OS cells transfected with empty vector (vec) or HA-tagged Praf2. The photographs are representative of the two main GFP-Bax localisations observed in the samples: diffuse cytosolic and aggregated. Above the photographs are indicated the % of GFP positive cells with an aggregated localisation after transfecting cells with empty vector or HA-tagged Praf2 in two independent experiments.

It is widely accepted that elevated Bcl-xL protein levels decrease susceptibility to apoptosis by binding and inactivating pro-apoptotic proteins [8]. Furthermore it has been reported that overexpression of Yip3p (yeast Pra1/Rabac1 homologue) severely inhibited cell growth [38]. We therefore tested if Praf2 overexpression could induce cell death, and if this could be blocked by the concomitant expression of Bcl-xL or the Bcl-xLΔTM mutant that was shown to be unable to bind to Praf2. HeLa cells were transfected with vector alone, with Praf2 or co-transfected with Praf2 and Bcl-xL or Praf2 and Bcl-xLΔTM. Fig. 6A shows that Praf2 transfection resulted in a strong induction of cell death, with almost 65% of cells becoming PI positive. Interestingly Praf2 induced cell death was completely inhibited by the concomitant transfection of full length Bcl-xL, but no inhibition was observed when Praf2 was cotransfected with the Bcl-xLΔTM mutant. Furthermore, cell death induced by Praf2 is accompanied by caspase activation as assessed by appearance of the cleaved form of PARP. Again, this phenomenon was completely blocked by Bcl-xL but not by the Bcl-xLΔTM mutant (Fig. 6B). Inhibition of Praf2-induced PARP cleavage was also prevented by the concomitant expression of Bcl-2 (data not shown).

Next, we wanted to assess if Praf2-induced apoptosis involved the translocation of Bax to the mitochondria, an early event in the intrinsic apoptotic pathway. We therefore cotransfected a GFP-Bax construct with Praf2 or vector control in U2OS. Fig. 6C shows that GFP-Bax expression in U2OS cells can have either a diffuse cytosolic staining, or can be aggregated in clusters. We have previously shown that GFP-Bax aggregation, in the same cell line, was coincident with mitochondrial dysfunction, as measured by a loss of ΔΨm [39]. We used an excess of Praf2-expressing or empty (Vec) plasmids in order to obtain that all cells receiving GFP-Bax also received Praf2 and counted the % of transfected cells with diffused or aggregated GFP-Bax signal. The result presented in Fig. 6C shows that Praf2 co-transfection was associated with more than 30% of cells displaying a GFP-Bax aggregated staining, respect to only 2% observed in empty-vector co-tansfected cells.

### Knock-down of Praf2 increases cellular survival

**Figure 7:**
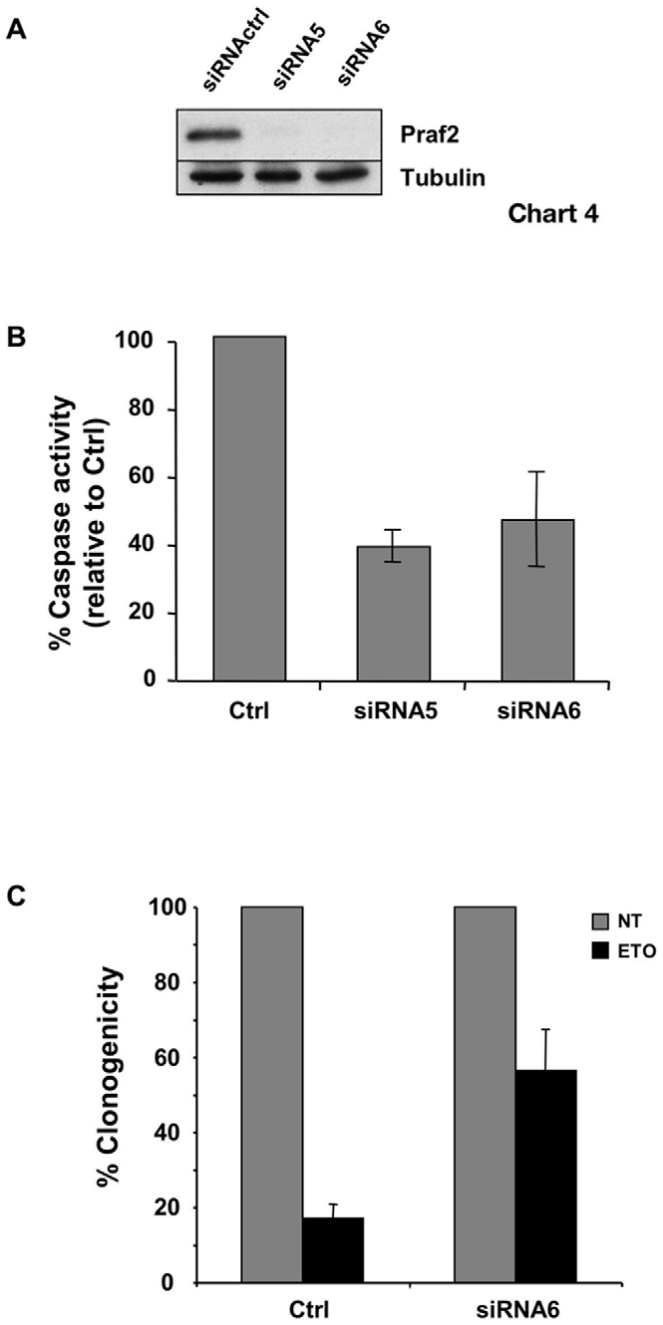
Praf2 knock down reduces apoptosis and increases clonogenicity of U2OS cells treated with etoposide. (**A**) Analysis by immunoblot of the efficacy of Praf2 knock down following transfection of U2OS cells with two different siRNAs targeting the Praf2 mRNA. (**B**) U2OS cells transfected with control (Ctrl) or Praf2 targeting siRNAs (siRNA5 and siRNA6) after 48 hours were treated with 50 µM etoposide for 24 hours. Cellular caspase 3 and 7 activities were measured using the Caspase-Glo 3/7 luminometric assay. The graph show the percentage of caspase 3 and 7 activation in samples treated with the Praf2 targeting siRNAs compared with the activity present in cells transfected with the control siRNA in 3 independent experiments. (**C**) Clonogenicity of U2OS cells transfected with control or Praf2 targeting siRNAs and treated or not with etoposide. 48 hours after transfection of U2OS cells with control siRNA or Praf2 siRNA6, cells were treated with 50 µM etoposide for 3 hours and than shifted again to normal medium for 2 weeks. Colonies were colored using crystal violet. The graph represents the percentage of colonies grown after etoposide treatment respect to the untreated control.

We next wanted to define if reduction of Praf2 expression by RNA interference could also influence cellular viability. The osteosarcoma cell line U2OS expresses relatively high levels of Praf2. We knocked-down Praf2 expression in U2OS cells using two different siRNAs targeting different regions of human Praf2 mRNA. Western blot analysis of Praf2 shows a strong decrease in Praf2 expression with respect to control-transfected cells (Fig. 7A). No obvious morphological difference was discernible between control and Praf2 knock-down cells 72 hours after transfection in normal growth conditions. We therefore asked if Praf2 silencing could affect cellular sensibility to the toxic effect of a chemotherapeutic agent like etoposide. As shown in Fig. 7B silencing of Praf2 with both the siRNAs chosen resulted in a reduction of more than 50% in caspase activation relative to control transfected cells. Accordingly, clonogenicity of U2OS cells treated with etoposide increased from below 20% of Ctrl transfected cells to almost 60% in Praf2 silenced cells (Fig. 7C). The decrease in caspase activation observed after Praf2 RNAi was not apoptotic stimulus-specific nor cell type-specific, as it was evident also in U2OS cells treated with paclitaxel or doxorubicin (Fig. S2A) and in the breast cancer cell line MDA-MB 231 treated with etoposide (Fig. S2B).

## Discussion

Bcl-xL is a C-tail anchored protein with the ability to localise on several intracellular membranes. In order to have a wide picture of the set of membrane proteins interacting with Bcl-xL, we performed Tandem Affinity Purification from total cellular membranes of HeLa cells stably expressing TAP-tagged Bcl-xL. Given that nonionic detergents like Triton-100 or NP-40 are known to alter the conformation of proteins of the Bcl-2 family [23], all the experiments were performed in presence of CHAPS, a detergent that leaves the conformation of Bcl-xL unchanged. As listed in Table 1, we found many proteins co-purifying with TAP-Bcl-xL. However, those are likely to reflect true protein-protein interactions for at least two reasons. First of all, as shown in lane 1 of Fig. 2C, purifications from the same cell line carrying a TAP-tag-only construct recover almost undetectable proteins, suggesting that all recovered proteins are indeed associated to Bcl-xL. Second, as discussed below, using this procedure we found co-eluting with Bcl-xL many proteins known to be able to specifically interact with Bcl-xL. We have sub-divided the list of proteins found to interact with Bcl-xL into the functional categories discussed below. It is worth to notice that all the proteins found are either trans-membrane proteins or soluble proteins localised in intracellular organelles. Most of them localise to the sub-cellular compartments known to be targeted by Bcl-xL. Many of them are multiple components of protein complexes. These observations constitute a good indication of the validity of the approach used and of the specificity and sensitivity of the technique used. We can therefore draw the conclusion that Bcl-xL can be engaged in several different protein complexes at several sub-cellular sites. This conclusion is further supported by the sucrose gradient fractionation data presented in Fig. 1, showing a spread distribution of Bcl-xL over a wide range of molecular weights, without discrete peaks of sedimentation.

### Bcl-2 family members

The list of proteins found to interact with Bcl-xL, shown in Table 1, includes the multidomain Bcl-2 family members Bax and Bak, together with the BH3-only proteins Bim, Puma, Bid and Bad. Strikingly, these are exclusively members of the subset of BH3-only proteins shown to have highest affinity for Bcl-xL in recent binding studies [40]. The presence of Bax indicates that perhaps mitochondrially localised Bax can be bound by Bcl-2 pro-survival proteins in an analogous manner as that now demonstrated conclusively for Bak [41]. BH3-only proteins are low abundance proteins and of small molecular weight, therefore making them difficult to detect by mass spectrometry. The fact that these proteins were detected at levels allowing protein sequencing, and in concordance with recently published literature, indicates the purifications were working reliably and efficiently.

### Mitochondrial energy metabolism and physiology

The main mitochondrial protein complex co-purifying with Bcl-xL was the F_1_F_0_ ATP Synthase complex. The F_1_F_0_ ATP Synthase complex is a highly conserved enzyme complex catalysing the terminal step in ATP synthesis. The mammalian complex contains at least 17 subunits, 10 of which are present in the list of Bcl-xL interactors (Table 1). The functional significance of a direct biochemical interaction between Bcl-xL and the ATP Synthase is presently unclear. It could be that what we observe is not a direct interaction but is mediated by Bax, given that Bax is interacting with Bcl-xL in our experimental setting, and given that the F_1_F_0_ ATP Synthase complex is found also to interact with Bax in TAP-Bax purifications (J.R.C and J.D unpublished observations). It is interesting to note that changes in ΔΨm occurring at the time of onset of apoptosis do not have a defined molecular mechanism. In the absence of sufficient flux through the electron transport chain hydrolysis of ATP via reversal of the F_1_F_0_ ATP Synthase could provide an alternative mechanisms by which ΔΨm can be maintained. It is possible that Bax and Bcl-xL have as yet undefined roles in coordinating (Bcl-xL) or disrupting (Bax) the metabolic mechanisms by which ATP production and maintenance of ΔΨm are coordinated.

### Trafficking and transporters

Our screen for Bcl-xL interacting proteins unexpectedly revealed the ability of Bcl-xL to interact with several proteins with a known or putative role in the secretory branch of intracellular trafficking. Examples of possible connections between protein secretion and apoptosis do exist in the literature. DAD1, the smallest subunit of the Ribophorin/OST complex, catalysing the first step in N-linked glycosylation of proteins, was named after it was originally identified as Defender Against apoptotic cell Death [42]. DAD1 has also been reported to interact with Mcl-1 in a yeast two hybrid assay [43]. These interactions may underly a link between glycosylation defects and the initiation of apoptosis, as suggested in the work of Hauptmann et al. [44]. The authors report that inhibition of N-linked glycosylation in yeast results in a range of phenotypes similar to those seen during apoptotic cell death of mammalian cells, such as DNA fragmentation and phosphatidylserine exposure, and these could be blocked by Bcl-2. We speculate that the ability of Bcl-2/xL to counteract the loss of viability due to glycosylation defects may be mediated by the novel interactions we identified.

An ability of Bcl-xL to modulate the secretory pathway would fit well with the view of Bcl-xL as a general guardian of cellular viability. Often cellular receptors are able to transduce intracellular survival signals and soluble secreted factors are mediators of growth/survival signals. The same is true for nutrient transporters that need to be heavily modified by the secretory pathway in order to be correctly delivered to the membrane. Is worth to notice that most of the transporters we found associated to Bcl-xL have been previously implicated in the regulation of cellular viability. The amino acid transporter SLC3A2, for instance, is a cell surface protein that is also able to associate with integrins and to mediate growth/survival signals [45]. Similarly, inhibition of the Na+/K+ transporter ATPase ATP1A1 was recently shown to induce sensitization to anoikis in cancer cells [46]. In general it has been demonstrated that, once on the membrane, nutrient transporters increase cell viability in nutrients limiting conditions [47]. Interestingly the work of Erdinger et al. shows that an increased persistence of nutrient transporters on the membrane can be achieved also by inhibiting the activity of Rab7 with the consequence of reducing endocytosis-associated transporter degradation. Given that Rab7 is among the Bcl-xL interacting proteins we have found (Table 1), we can speculate that Bcl-xL could have evolved a complex strategy to increase cellular viability at the membrane level: increase the efficiency of receptor/transporter delivery to the membrane, acting on the secretory pathway and decrease their turnover modulating Rab7 activity.

Finally, the interactions we found may also not be directly related to the anti-apoptotic role of Bcl-xL. Recently several reports have suggested a positive role for Bcl-xL in cellular invasiveness and tumor metastasis, independent of its ability to protect from cell death [9]–[11]. Modulation of the secretory pathway could be related to adhesion, motility and invasion, given that all the molecular players in these processes are either soluble secreted proteins (chemokines) or membrane receptors that need to be folded, modified and delivered by the secretory pathway.

### Praf2

Given the presence of so many proteins with a role in the secretory branch of intracellular trafficking, we decided to validate the novel Bcl-xL interacting proteins found, focusing on Praf2, a small transmembrane protein with a putative role in ER to Golgi transport [26]. Praf2 belongs to the family of Prenylated Rab Acceptor (PRA) proteins. Vertebrates have three PRA family members, Rabac1, Praf2 and Arl6IP5. They are small trans-membrane proteins located to the Golgi complex (Rabac1) [48] and the Endoplasmic Reticulum (Praf2 and Arl6IP5) [33]. The degree of sequence homology among the PRA proteins is stronger between Praf2 and Arl6IP5 (45% of sequence identity) than between Rabac1 and Praf2 (25% of sequence identity). The cellular function of PRA proteins has not been univocally defined. It has been suggested that Rabac1 could mediate loading of the inactive-GDI-bound cytosolic pool of Rabs on intracellular membranes, given its *in vitro* GDI displacement activity [49]. Surprisingly though, yeast mutants deleted of the yip3 gene (*S.cerevisiae* Rabac1 homologue) did not show any changes in the intracellular localization of Rabs. Instead a striking proliferation of the ER membranes, accompanied by inhibition of cell growth, was observed following yip3 overproduction [38].

Rabac1 has been shown to interact with BHRF1, a viral homologue of Bcl-2. Interestingly the anti-apoptotic activity of BHRF1 was reduced upon interaction with Rabac1 [32]. Furthermore it has been shown that Praf2 expression correlated with cerulenin-induced apoptosis [50] and Arl6IP5 is a key mediator of the apoptosis induced by C/EBPalpha [51]. We speculated therefore that the interaction of Bcl-xL with Praf2 might constitute a link between the secretory and the apoptotic pathways.

In this study, we have confirmed the ability of Praf2 to interact with Bcl-xL and defined that the interaction depends on Bcl-xL's C-terminal transmembrane (TM) region. The dependency on the TM region might be interpreted in different ways: either the TM region is directly involved in the interaction or is a pre-requisite for the interaction because it is the anchor that localises Bcl-xL on the membrane were it can find its partner Praf2. Given that secondary structure prediction algorithms suggest that Praf2 is almost entirely embedded in the membrane bilayer, it is reasonable to think that the interaction could be directly mediated by a hydrophobic amino-acid stretch like the TM domain.

Unlike Bcl-2, that is predominantly located to the ER, Bcl-xL has been suggested to be mainly a mitochondrial protein located at the Mitochondrial Outer Membrane (MOM) [52]. Praf2, instead, has been shown to be mainly located at the ER (data not shown and [33]). How can two proteins located at different intracellular compartments interact? In our cellular fractionation experiments we can always observe a small fraction of Bcl-xL located to the microsomal light membrane fraction (Fig. 2 and data not shown). One possibility is that the interaction is limited to the small fraction of ER-based Bcl-xL. We do observe, in fact, a partial co-localization between Bcl-xL and Praf2 particularly pronounced in the cellular perinuclear region (Fig. S3). Another possibility is that the localisation of one or both proteins is dynamic and the interaction is limited to a particular physiological stage. It is known for instance that Bcl-xL translocates from the cytosol to the mitochondria following an apoptotic stimulus [23]. There could be a yet undefined stimulus that induces Bcl-xL relocalisation mainly to the ER. One can even hypothesize that Praf2 would function as the receptor of Bcl-xL under such a condition. A final possibility is that the most relevant physiological interaction occurs between Praf2 and Bcl-2 (more than Bcl-xL), given that Bcl-2 localises mainly to the ER [52]. We have indeed demonstrated that Praf2 is able to interact with both Bcl-2 and Bcl-xL (Fig. 4).

Independently to which is the relevant physiological partner, our study shows that modulation of Praf2 expression could affect cellular viability. We found that overexpression of Praf2 induces translocation and aggregation of Bax at the mitochondria and, ultimately, increases cellular caspase activity and cell death (Fig. 6). On the same track, reducing Praf2 expression by RNAi in the U2OS osteosarcoma cell line, we observed an increased resistance to cytotoxic effect of the chemotherapeutic drug etoposide (Fig. 7). What could be the mechanism explaining Praf2's proapoptotic activity? And how to reconcile these data with the observation that the expression of Praf2 in human tumor tissues is higher than in normal tissues of the same patient? [53] Another group of ER based proteins found to interact with Bcl-xL and/or Bcl-2 and to modulate cell death is composed of members of the Reticulon family of proteins: RTN1-C, RTN4A and RTN3. Interestingly the yeast homologue of Praf2, Yip3p, has been found interacting with the reticulon Rtn1p [38], and our preliminary results suggest that Praf2 is also able to interact with RTN4 (data not shown). The mechanism by which RTN proteins modulate sensitivity to apoptosis is also not entirely clear but is supposed to be based on the ability of RTN proteins to influence Bcl-xL/Bcl-2 subcellular localisation. RTN4 has been suggested to reduce the antiapoptotic activity of Bcl-xL and Bcl-2 by forcing their localisation onto the ER and reducing their presence on the mitochondria [54]. RTN3, instead, was shown to be able to increase the accumulation of Bcl-2 on mitochondria [55]. Interestingly, although RTN3 expression induces apoptosis, its concomitant expression with Bcl-2 increases Bcl-2's antiapoptotic activity [56]. One possibilty is that Praf2 and RTN4 work in the same multiprotein complex having the ability to define the subcellular localisation of Bcl-2 proteins. In this scenario, the consequences of Praf2 overexpression on cellular viability could vary depending on the type of anti-apoptotic Bcl-2 protein expressed and on their dependency for survival. We know for instance that U2OS cells strongly rely on Blc-xL for survival, because Bcl-xL RNAi in U2OS rapidily triggers apoptosis (data not shown). If Praf2 would sequester a pool of cellular Bcl-xL on the ER, the consequence of Praf2 RNAi in U2OS could be a shift of the same pool to the mitochondria where it could contribute to protection against apoptotic stimuli that trigger mitochondria destabilization. This is exactly what we observe in U2OS cells treated with etoposide (Fig. 7).

The possibility that Praf2, forming a complex with RTN proteins could be able to potentiate the anti-apoptotic activity of Bcl-2/xL, as shown for RTN3 [56], could also help to explain why an increased Praf2 expression would be selected during tumor formation. The negative effect of Praf2 on cellular viability would be counterbalanced by the concomitant increase in cellular survival potential once anti-apoptotic oncogenes like Bcl-2 and/or Bcl-xL becomes activated.

### Conclusions

In conclusion, we have identified a number of novel Bcl-xL associated proteins involved in several biological processes. The variety of proteins found to interact with Bcl-xL could explain the pleiotropic effect of Bcl-xL overexpression. Characterisation of Praf2, one of the novel Bcl-xL interacting proteins, revealed that modulation of its expression could interfere with cellular sensitivity to chemotherapy. We speculate therefore that some of the novel interactions described here could be at the basis of the unique role played by Bcl-xL in cellular resistance to cytotoxic agents.

## Materials and Methods

### Reagents and cell culture

U2OS and HEK 293T cells were described previously [28]. HeLa cells were obtained from Dr. MV Ursini (Institute of Genetics and Biophysics). Etoposide, colloidal Coomassie, monoclonal anti-HA conjugated Agarose (clone HA-7) and monoclonal anti Flag (M2) were from Sigma-Aldrich. Monoclonal antibodies directed against PARP, Cytochrome c (7H8.2C12) and polyclonal against Bcl-xL were from BD Biosciences, polyclonal against Rab4 was from Abcam, was from Sigma, monoclonal anti Bax (2D2), polyclonal anti Sec23 (E19) and monoclonal anti HA (F7) were purchased from Santa Cruz Biotechnology. An antibody against Praf2 was obtained immunising rabbits with GST-tagged full-length recombinant Praf2.

HeLa, U2OS and HEK 293T cells were cultured in Dulbecco's modified Eagle's medium supplemented with 100 µg/ml streptomycin, 100 units/ml penicillin, and 10% fetal bovine serum (FBS) (pH 7.2–7.4) in a humidified atmosphere containing 5% CO_2_ at 37°C.

### Gene Silencing

Control and Praf2-targeting siRNA oligonucleotides were from Qiagen. The sequences used (only sense sequences given) were: Praf2 siRNA5, 5′-CCAGGUCAAGACAUUGCCAAAdTdT-3′; Praf2 siRNA6, 5′-GUGUUGCUGCAACAAUAAAdTdT-3′. siRNAs were transfected with the “fast-forward” protocol using HiPerFect (Qiagen) as transfection reagent. Briefly, 2×10^5^ cells/well were seeded in 6-well plates and incubated with the transfection complexes (20 nM siRNA and 12 µl of HiPerFect in cell growth medium). After 48–72 hours cells were processed.

### Western blotting

Whole cells or membrane fractions were lysed in CHAPS Extraction Buffer (CEB) [50 mM Tris/Hcl pH 8.0, 150 mM NaCl, 5% glycerol, 1% CHAPS, 2 mM EGTA, 1 mM DTT, 1×Complete™ (Roche)]. After 15 min incubation on ice, lysates were cleared by centrifugation. Total cell extracts were separated on 10% or 12.5% SDS-PAGE and transferred to Immobilon-P transfer membranes (Millipore). After blocking with 5% non-fat milk membranes were incubated with primary antibodies as recommended by the supplier. The immune complexes were detected by the ECL detection system according to the manufacturer's protocol (Millipore).

### Sucrose gradients

12×10 cm^2^ confluent dishes of U2OS cells were collected by trypsinisation, washed with PBS and resuspended in 1.5 ml of CHAPS Extraction Buffer (CEB) [50 mM Tris/Hcl pH 8.0, 150 mM NaCl, 5% glycerol, 1% CHAPS, 2 mM EGTA, 1 mM DTT, 1× Complete™ (Roche)]. Lysates were cleared by centrifugation (15 min at 13200 rpm) and 1 ml was loaded on the top of a step gradient obtained by layering 2 ml of each of the following sucrose solutions: 40 - 32.5 – 25 - 17.5 and 10% sucrose in 50 mM Tris/Hcl pH 8.0, 150 mM NaCl, 1% CHAPS, 2 mM EGTA, 1 mM DTT. The gradient was centrifuged at 35.000 rpm for 22 hours in a SW41 rotor (Beckman), with break at position zero. 60 fractions (4 drops/fraction) were collected from the bottom of the tube after piercing the tube with a hot needle. Equal volumes (30 µl) of eve numbers fractions were analysed by Western blotting as described.

### Subcellular fractionation

4×10 cm^2^ confluent dishes of HeLa/TAP-Bcl-xL were collected by trypsinisation, washed with PBS and resuspended in HS buffer (10 mM HEPES pH7.4, 250 mM sucrose, 1× Complete™). Cells were lysed by Dounce homogenisation (30 strokes, pestle B) and centrifuged 10 min at 600×g to pellet the nuclear fraction (Nuclei). The supernatant was centrifuged 15 min at 9,000×g to pellet the heavy membrane fraction (HMM). The supernatant was centrifuged 30 min at 100,000×g to pellet the light membrane fraction (LMM). The final supernatant was taken as S100 fraction. Nuclei, HMM and LMM pellets were resuspended in CHAPS Extraction Buffer (CEB) [50 mM Tris/Hcl pH 8.0, 150 mM NaCl, 5% glycerol, 1% CHAPS, 2 mM EGTA, 1 mM DTT, 1× Complete™ (Roche)] and equal amount of proteins were analysed by Western blotting as described above.

### Generation of TAP-Bcl-xL expressing cells and Tandem Affinity Purification

A pcDNA3 based expression construct designed to fuse the TAP tag with an optimised TEV cleavage consensus sequence to the N-Terminus of Bcl-xL was created. The construct had in series: a Kozak initiation site, a 3 Gly spacer, 2 IgG tags, a TEV spacer (DYDIPTT) and a TEV cleavage site (ENLYFQ*G), a 5 Gly spacer, a CBP tag and a 6 Gly spacer. A control construct was created by inserting a stop codon after the TAP tag sequence. After electroporation, HeLa cells clones stably expressing the fusion protein or the TAP alone, were selected using 0.8 mg/ml of G418.

TAP purifications were performed as previously describe [28] with some modifications. Briefly, HeLa cells expressing TAP-Bcl-xL or TAP-stop were adapted to grow in supension in glass spinning bottles. Cell pellets from 1.5–2 liters of cultures were lysed on ice by dounce homogenization in 10 ml of HS buffer (10 mM HEPES pH7.4, 250 mM sucrose, 1× Complete™). Total membranes where isolated by centrifugation (16000×g for 30 mins) and extracted with 10 ml of CHAPS Extraction Buffer (CEB) [50 mM Tris/Hcl pH 8.0, 150 mM NaCl, 5% glycerol, 1% CHAPS, 2 mM EGTA, 1 mM DTT, 1× Complete™ (Roche)]. The clarified membrane extract was incubated with 600 µl of a 50% suspension of pre-washed IgG-linked agarose (Sigma) for 2 hrs at 4°C with rotation. The agarose matrix was then washed with 30 ml of CEB and 10 ml of TEV cleavage buffer (10 mM Tris HCl pH 8.0, 150 mM NaCl, 0.1% CHAPS, 0.5 mM EDTA, 1 mM DTT). The agarose matrix was then incubated with 950 µl TEV cleavage buffer and 500 U TEV protease (Invitrogen) for 2 hrs at RT, followed by O/N at 4°C with rotation. The resulting eluate was supplemented with 3.5 ml calmodulin binding buffer (10 mM Tris HCl pH 8.0, 150 mM NaCl, 0.1% CHAPS, 1 mM Mg acetate, 1 mM imidazole, 2 mM CaCl2, 10mM BME) and 3.5 µl 1 M CaCl_2_ and incubated with 500 µl of a 50% suspension of Calmodulin-linked affinity resin (Stratagene) for 4 hrs at 4°C with rotation. The Calmodulin resin was washed 30 ml of binding buffer and the bound proteins eluted with 5×200 µl fractions of calmodulin elution buffer (2 mM CaCl_2_ was substituted for 2 mM EGTA). TAP eluates were concentrated using Microcon YM-10 columns (Millipore) to a final volume of ∼30 µl and purified proteins visualized on 4–12% NuPAGE gels (Invitrogen) using colloidal coomassie (Sigma).

### Mass spectrometry

Bands corresponding to the most abundant protein species were excised from SDS PAGE gels and digested with 16 Units Porcine trypsin (Promega) according to the manufacturer's instructions. Peptides were extracted 3 times with 30 µl 5% formic acid, dried in a SpeedVac and desalted. Immediately prior to analysis peptides were resuspended in 7 µl 1% formic acid and 5 µl injected onto a nano HPLC system (LC Packings) with 75 µm I.D.×150 mm column packed with Vydac C18 (5 µm particles). Separation was achieved using a flow rate of 250 nl/min (gradient 0% to 95% of 0.1% acetic acid in acetonitrile) and eluted peptides were analyzed directly by tandem electrospray mass spectrometry using either a qTOF (Micromass) or ABI 4000 QTRAP MS/MS System (Applied Biosystems). The resulting mass spectra were queried against the NCBI non-redundant database using the Mascot software (Matrix Science). One missed cleavage per peptide and an initial mass tolerance of 10 ppm were used in all searches.

### Immunoprecipitations

HEK 293T cells (1×10^5^/35cm^2^ dishes) were transfected using Polyfect as transfection reagent (Qiagen). After 24 hours cells were scraped on dish, washed with PBS and lysed in 500 µl of CEB [50 mM Tris/Hcl pH 8.0, 150 mM NaCl, 5% glycerol, 1% CHAPS, 2 mM EGTA, 1 mM DTT, 1× Complete™ (Roche)]. Lysates were cleared at 13000 rpm for 15 min and therefore incubated with 20 µl of anti HA conjugated Agarose (Sigma), previously equilibrated in CEB, for 2 hours at 4°C, rotating. Agarose was washed 3 times with 1 ml of CEB, resuspended in 30 µl of 2× Laemmli buffer and boiled 5 min. Equal volumes of supernatants were resolved on 12% SDS-PAGE and analysed by Western blot.

For the immunoprecipitation of endogenous Bcl-xL from U2OS cells, 2×10 cm^2^ dishes of 60% confluent U2OS were transfected using Effectene (Qiagen) with 2 µg of either empty pcDNA3C3xHA or pcDNA3C3xHA/Praf2. After 24 hours cells are extracted with 800 µl of CEB and cleared by centrifugation. 560 µg of protein extracts were incubated with 20 µl of anti HA conjugated Agarose (Sigma), previously equilibrated in CEB, for 2 hours at 4°C, rotating. Agarose was washed 3 times with 1 ml of CEB, resuspended in 30 µl of 2× Laemmli buffer and boiled 5 min. Equal volumes of supernatants were resolved on 12% SDS-PAGE and analysed by Western blot.

### Cell viability assays

Caspase-3 and -7 activities were measured by using a Caspase-Glo 3/7 Assay kit (Promega). Experiments were carried out following manufacturer's recommended procedures. Briefly, 5×10^3^ of U2OS cells/well were plated in white-walled 96-well plates and transfected the same day with 20 nM of the indicated siRNAs using HiPerfect (Qiagen). After treating the cells as specified in figure legends, the Caspase-Glo 3/7 reagent was added to the wells followed by measurements of luminescence 1 h later with a plate-reading luminometer (GloMax® 96 Luminometer, Promega). To measure clonogenicity, U2OS cells were transfected with control or Praf2 targeting siRNAs as specified above and after 24 hours plated in 10 cm^2^ dishes in triplicate at 3000 cells/dish density. The day after cells were treated with etoposide 50 µM for 3 hours and then medium was changed. Cells were grown for 10–15 days and colonies were stained using crystal violet.

### Immunofluorescence

U2OS cells, plated on glass coverslips were co-transfected with GFP-Bax and 3xHA-Praf2 or the empty vector using Effectene (Qiagen) as transfection reagent. After 22 hours cells were fixed in 4% paraformaldehyde in PBS for 10 min at room temperature and washed three times in PBS. Paraformaldehyde was quenched with 10 mmol/L ammonium chloride, and cells were permeabilized with PBS, 0.1% Triton X-100 for 5 min at room temperature. The coverslips were washed three times in PBS and then blocked in PBS, 0.5% bovine serum albumin for 30 min. Cells were incubated with anti HA monoclonal antibody diluted in PBS, 0.5% bovine serum albumin for 1 h at room temperature. Coverslips were washed five times in PBS and treated for 1 h at room temperature with the secondary anti mouse antibody AlexaFluor 594 (Invitrogen) diluted in PBS, 0.5% bovine serum albumin. Coverslips were washed in PBS and mounted with ProLong Gold anti fade reagent with DAPI (Invitrogen). The cells were visualized by fluorescence microscopy (Leica DM6000).

## Supporting Information

Figure S1 **Analysis of the expression levels of TAP-Bcl-xL, Bcl-xL and Praf2 in HeLa-TAP-Bcl-xL and U2OS cells.** Equal amount of total cell lysates were resolved on SDS-PAGE and analysed by Western blotting using the antibodies indicated. Tubulin was used as loading control.
(TIF)

Figure S2 **Praf2 knock down reduces chemotherapy-induced apoptosis in U2OS and MDA-MB 231cells.** (**A**) Praf2 RNAi in U2OS reduces apoptosis induced also by paclitaxel and doxorubicin. Cells were transfected with control or Praf2 targeting siRNAs. After 48 hours cells were treated for 24 hours either with 100 nM paclitaxel (Sigma) or 0.5 µg/ml doxorubicin (Sigma). (**B**) Praf2 RNAi reduces etoposide-induced apoptosis also in MDA-MB 231cells. Cells were transfected with control or Praf2 targeting siRNAs. After 48 hours cells were treated for 24 hours with 50 µM etoposide. Cellular caspase 3 and 7 activities were measured using the Caspase-Glo 3/7 luminometric assay. The graph show the percentage of caspase 3 and 7 activation in samples treated with the Praf2 targeting siRNAs compared with the activity present in cells transfected with the control siRNA in 2 independent experiments.
(TIF)

Figure S3 **Praf2 partially co-localises with Bcl-xL in U2OS cells.** U2OS cells were transfected with HA-Praf2 and analysed by confocal immunofluorescence microscopy using an antibody directed against Bcl-xL (Pharmingen) and an antibody recognising the HA-tag (Sigma).
(TIF)

## References

[1] Iaccarino I, Hancock D, Evan G, Downward J (2003). c-Myc induces cytochrome c release in Rat1 fibroblasts by increasing outer mitochondrial membrane permeability in a Bid-dependent manner.. Cell Death Differ.

[2] Dong Z, Wang J (2004). Hypoxia selection of death-resistant cells. A role for Bcl-X(L).. J Biol Chem.

[3] Kauffmann-Zeh A, Rodriguez-Viciana P, Ulrich E, Gilbert C, Coffer P (1997). Suppression of c-Myc-induced apoptosis by Ras signalling through PI(3)K and PKB.. Nature.

[4] Amundson SA, Myers TG, Scudiero D, Kitada S, Reed JC (2000). An Informatics Approach Identifying Markers of Chemosensitivity in Human Cancer Cell Lines.. Cancer Res.

[5] Youle RJ, Strasser A (2008). The BCL-2 protein family: opposing activities that mediate cell death.. Nat Rev Mol Cell Biol.

[6] Desagher S, Osen-Sand A, Nichols A, Eskes R, Montessuit S (1999). Bid-induced conformational change of Bax is responsible for mitochondrial cytochrome c release during apoptosis.. J Cell Biol.

[7] Marani M, Tenev T, Hancock D, Downward J, Lemoine NR (2002). Identification of novel isoforms of the BH3 domain protein Bim which directly activate Bax to trigger apoptosis.. Mol Cell Biol.

[8] Adams JM, Cory S (2007). The Bcl-2 apoptotic switch in cancer development and therapy.. Oncogene.

[9] Fernández Y, España L, Mañas S, Fabra A, Sierra A (2000). Bcl-xL promotes metastasis of breast cancer cells by induction of cytokines resistance.. Cell Death Differ.

[10] Du YN, Lewis BC, Hanahan D, Varmus H (2007). Assessing Tumor Progression Factors by Somatic Gene Transfer into a Mouse Model: Bcl-xL Promotes Islet Tumor Cell Invasion.. PLoS Biology.

[11] Martin SS, Ridgeway AG, Pinkas J, Lu Y, Reginato MJ (2004). A cytoskeleton-based functional genetic screen identifies Bcl-xL as an enhancer of metastasis, but not primary tumor growth.. Oncogene.

[12] Vander Heiden MG, Choy JS, VanderWeele DJ, Brace JL, Harris MH (2002). Bcl-x(L) complements Saccharomyces cerevisiae genes that facilitate the switch from glycolytic to oxidative metabolism.. J Biol Chem.

[13] Li C, Fox CJ, Master SR, Bindokas VP, Chodosh LA (2002). Bcl-X(L) affects Ca(2+) homeostasis by altering expression of inositol 1,4,5-trisphosphate receptors.. Proc Natl Acad Sci U S A.

[14] Li H, Chen Y, Jones AF, Sanger RH, Collis LP (2008). Bcl-xL induces Drp1-dependent synapse formation in cultured hippocampal neurons.. Proc Natl Acad Sci U S A.

[15] O'Reilly LA, Huang DC, Strasser A (1996). The cell death inhibitor Bcl-2 and its homologues influence control of cell cycle entry.. EMBO J.

[16] Shimizu S, Kanaseki T, Mizushima N, Mizuta T, Arakawa-Kobayashi S (2004). Role of Bcl-2 family proteins in a non-apoptotic programmed cell death dependent on autophagy genes.. Nat Cell Biol.

[17] Priault M, Hue E, Marhuenda F, Pilet P, Oliver L (2010). Differential dependence on Beclin 1 for the regulation of pro-survival autophagy by Bcl-2 and Bcl-xL in HCT116 colorectal cancer cells.. PLoS ONE.

[18] Berman SB, Chen Y, Qi B, McCaffery JM, Rucker EB (2009). Bcl-x L increases mitochondrial fission, fusion, and biomass in neurons.. J Cell Biol.

[19] Vander Heiden MG, Li XX, Gottleib E, Hill RB, Thompson CB (2001). Bcl-xL promotes the open configuration of the voltage-dependent anion channel and metabolite passage through the outer mitochondrial membrane.. J Biol Chem.

[20] Shimizu S, Narita M, Tsujimoto Y, Tsujimoto Y (1999). Bcl-2 family proteins regulate the release of apoptogenic cytochrome c by the mitochondrial channel VDAC.. Nature.

[21] Rong Y, Bultynck G, Aromolaran AS, Zhong F, Parys JB (2009). The BH4 domain of Bcl-2 inhibits ER calcium release and apoptosis by binding the regulatory and coupling domain of the IP3 receptor.. Proc Natl Acad Sci U S A.

[22] Oberstein A, Jeffrey PD, Shi Y (2007). Crystal structure of the Bcl-XL-Beclin 1 peptide complex: Beclin 1 is a novel BH3-only protein.. J Biol Chem.

[23] Hsu YT, Wolter KG, Youle RJ (1997). Cytosol-to-membrane redistribution of Bax and Bcl-X(L) during apoptosis.. Proc Natl Acad Sci U S A.

[24] Jeong S, Gaume B, Lee Y, Hsu Y, Ryu S (2004). Bcl-x(L) sequesters its C-terminal membrane anchor in soluble, cytosolic homodimers.. EMBO J.

[25] Antonsson B, Montessuit S, Sanchez B, Martinou JC (2001). Bax is present as a high molecular weight oligomer/complex in the mitochondrial membrane of apoptotic cells.. J Biol Chem.

[26] Ruggiero AM, Liu Y, Vidensky S, Maier S, Jung E (2008). The endoplasmic reticulum exit of glutamate transporter is regulated by the inducible mammalian Yip6b/GTRAP3-18 protein.. J Biol Chem.

[27] Rigaut G, Shevchenko A, Rutz B, Wilm M, Mann M (1999). A generic protein purification method for protein complex characterization and proteome exploration.. Nat Biotechnol.

[28] Martins LM, Iaccarino I, Tenev T, Gschmeissner S, Totty NF (2002). The serine protease Omi/HtrA2 regulates apoptosis by binding XIAP through a reaper-like motif.. J Biol Chem.

[29] Cheng EHY, Sheiko TV, Fisher JK, Craigen WJ, Korsmeyer SJ (2003). VDAC2 inhibits BAK activation and mitochondrial apoptosis.. Science.

[30] Kuo TH, Kim HR, Zhu L, Yu Y, Lin HM (1998). Modulation of endoplasmic reticulum calcium pump by Bcl-2.. Oncogene.

[31] Hájek P, Chomyn A, Attardi G (2007). Identification of a novel mitochondrial complex containing mitofusin 2 and stomatin-like protein 2.. J Biol Chem.

[32] Li LY, Shih HM, Liu MY, Chen JY (2001). The cellular protein PRA1 modulates the anti-apoptotic activity of Epstein-Barr virus BHRF1, a homologue of Bcl-2, through direct interaction.. J Biol Chem.

[33] Schweneker M, Bachmann AS, Moelling K (2005). JM4 is a four-transmembrane protein binding to the CCR5 receptor.. FEBS Lett.

[34] Muchmore SW, Sattler M, Liang H, Meadows RP, Harlan JE (1996). X-ray and NMR structure of human Bcl-xL, an inhibitor of programmed cell death.. Nature.

[35] Wang HG, Rapp UR, Reed JC (1996). Bcl-2 targets the protein kinase Raf-1 to mitochondria.. Cell.

[36] Denis GV, Yu Q, Ma P, Deeds L, Faller DV (2003). Bcl-2, via its BH4 domain, blocks apoptotic signaling mediated by mitochondrial Ras.. J Biol Chem.

[37] Minn AJ, Kettlun CS, Liang H, Kelekar A, Vander Heiden MG (1999). Bcl-xL regulates apoptosis by heterodimerization-dependent and -independent mechanisms.. EMBO J.

[38] Geng J, Shin ME, Gilbert PM, Collins RN, Burd CG (2005). Saccharomyces cerevisiae Rab-GDI Displacement Factor Ortholog Yip3p Forms Distinct Complexes with the Ypt1 Rab GTPase and the Reticulon Rtn1p.. Eukaryotic Cell.

[39] Cross JR, Postigo A, Blight K, Downward J (2008). Viral pro-survival proteins block separate stages in Bax activation but changes in mitochondrial ultrastructure still occur.. Cell Death Differ.

[40] Chen L, Willis SN, Wei A, Smith BJ, Fletcher JI (2005). Differential targeting of prosurvival Bcl-2 proteins by their BH3-only ligands allows complementary apoptotic function.. Mol Cell.

[41] Willis SN, Chen L, Dewson G, Wei A, Naik E (2005). Proapoptotic Bak is sequestered by Mcl-1 and Bcl-xL, but not Bcl-2, until displaced by BH3-only proteins.. Genes Dev.

[42] Kelleher DJ, Gilmore R (1997). DAD1, the defender against apoptotic cell death, is a subunit of the mammalian oligosaccharyltransferase.. Proc Natl Acad Sci U S A.

[43] Makishima T, Yoshimi M, Komiyama S, Hara N, Nishimoto T (2000). A subunit of the mammalian oligosaccharyltransferase, DAD1, interacts with Mcl-1, one of the bcl-2 protein family.. J Biochem.

[44] Hauptmann P, Riel C, Kunz-Schughart LA, Fröhlich K, Madeo F (2006). Defects in N-glycosylation induce apoptosis in yeast.. Mol Microbiol.

[45] Feral CC, Nishiya N, Fenczik CA, Stuhlmann H, Slepak M (2005). CD98hc (SLC3A2) mediates integrin signaling.. Proc Natl Acad Sci U S A.

[46] Simpson CD, Mawji IA, Anyiwe K, Williams MA, Wang X (2009). Inhibition of the sodium potassium adenosine triphosphatase pump sensitizes cancer cells to anoikis and prevents distant tumor formation.. Cancer Res.

[47] Edinger AL, Cinalli RM, Thompson CB (2003). Rab7 prevents growth factor-independent survival by inhibiting cell-autonomous nutrient transporter expression.. Dev Cell.

[48] Abdul-Ghani M, Gougeon PY, Prosser DC, Da-Silva LF, Ngsee JK (2001). PRA isoforms are targeted to distinct membrane compartments.. J Biol Chem.

[49] Sivars U, Aivazian D, Pfeffer SR (2003). Yip3 catalyses the dissociation of endosomal Rab-GDI complexes.. Nature.

[50] Geerts D, Wallick CJ, Koomoa DT, Koster J, Versteeg R (2007). Expression of prenylated Rab acceptor 1 domain family, member 2 (PRAF2) in neuroblastoma: correlation with clinical features, cellular localization, and cerulenin-mediated apoptosis regulation. Clin.. Cancer Res.

[51] Wang G, Shi X, Salisbury E, Timchenko NA (2008). Regulation of apoptotic and growth inhibitory activities of C/EBPalpha in different cell lines.. Exp Cell Res.

[52] Kaufmann T, Schlipf S, Sanz J, Neubert K, Stein R (2003). Characterization of the signal that directs Bcl-x(L), but not Bcl-2, to the mitochondrial outer membrane.. J Cell Biol.

[53] Fo CS, Coleman CS, Wallick CJ, Vine AL, Bachmann AS (2006). Genomic organization, expression profile, and characterization of the new protein PRA1 domain family, member 2 (PRAF2).. Gene.

[54] Tagami S, Eguchi Y, Kinoshita M, Takeda M, Tsujimoto Y (2000). A novel protein, RTN-XS, interacts with both Bcl-XL and Bcl-2 on endoplasmic reticulum and reduces their anti-apoptotic activity.. Oncogene.

[55] Wan Q, Kuang E, Dong W, Zhou S, Xu H (2007). Reticulon 3 mediates Bcl-2 accumulation in mitochondria in response to endoplasmic reticulum stress.. Apoptosis.

[56] Zhu L, Xiang R, Dong W, Liu Y, Qi Y (2007). Anti-apoptotic activity of Bcl-2 is enhanced by its interaction with RTN3.. Cell Biol Int.

