# Supplementary figures and images for: Praf2 Is a Novel Bcl-xL/Bcl-2 Interacting Protein with the Ability to Modulate Survival of Cancer Cells
Source: PLoS One. 2010 Dec 20;5(12):e15636. doi: 10.1371/journal.pone.0015636 (PMC3006391; doi:10.1371/journal.pone.0015636)

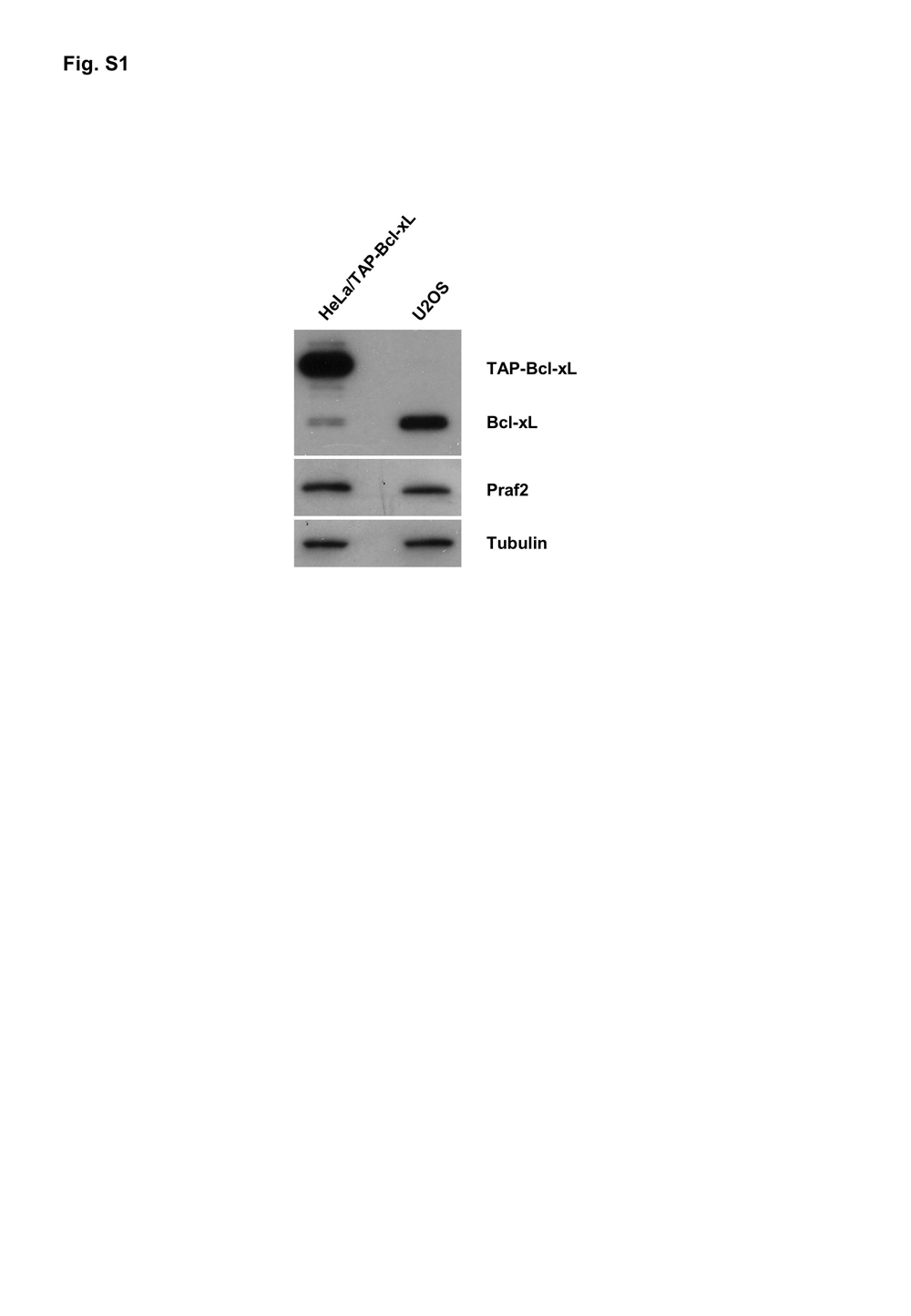

Supplement: Figure S1 — Analysis of the expression levels of TAP-Bcl-xL, Bcl-xL and Praf2 in HeLa-TAP-Bcl-xL and U2OS cells. Equal amount of total cell lysates were resolved on SDS-PAGE and analysed by Western blotting using the antibodies indicated. Tubulin was used as loading control. (TIF) [file pone.0015636.s001.tif]

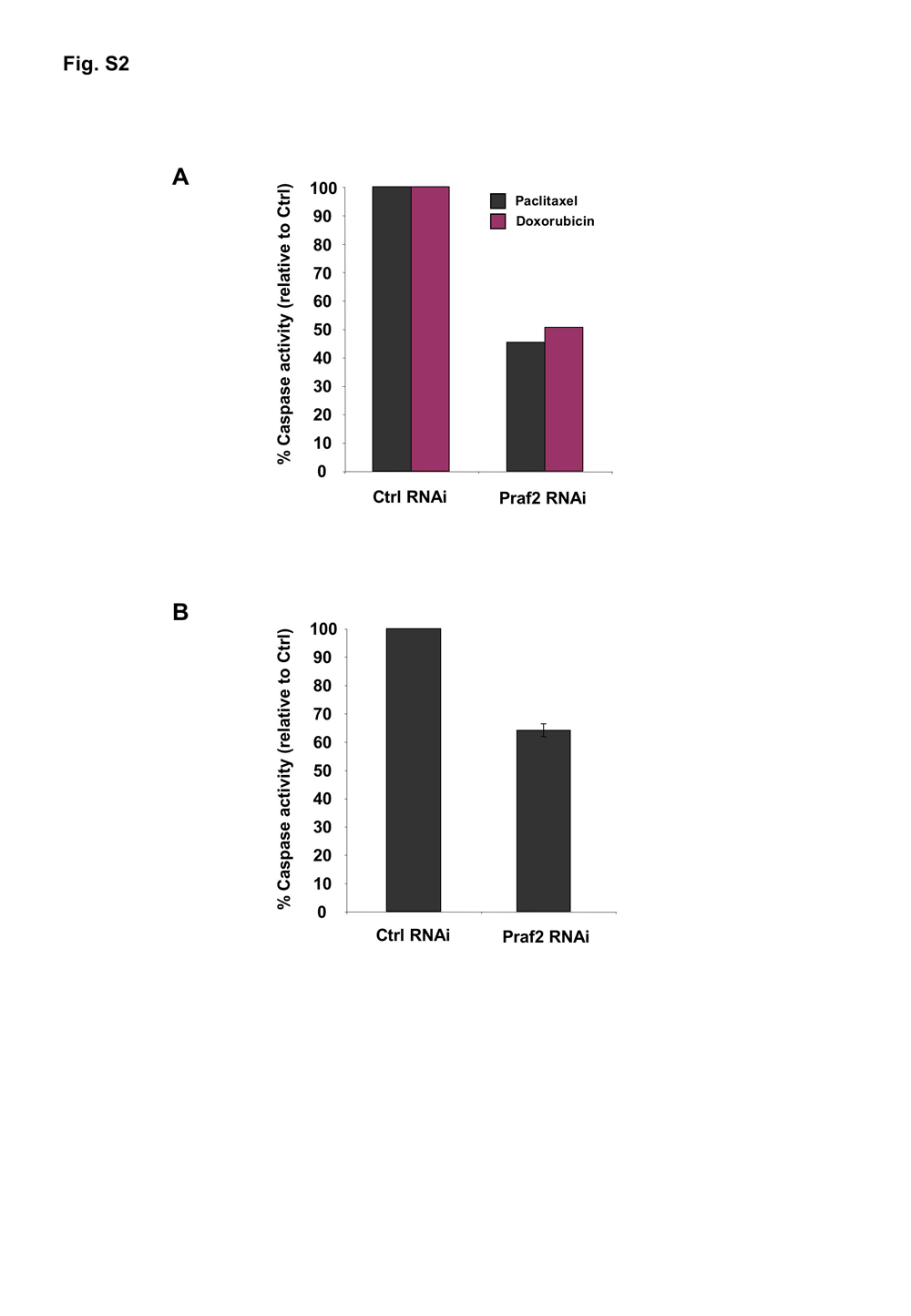

Supplement: Figure S2 — Praf2 knock down reduces chemotherapy-induced apoptosis in U2OS and MDA-MB 231cells. (A) Praf2 RNAi in U2OS reduces apoptosis induced also by paclitaxel and doxorubicin. Cells were transfected with control or Praf2 targeting siRNAs. After 48 hours cells were treated for 24 hours either with 100 nM paclitaxel (Sigma) or 0.5 µg/ml doxorubicin (Sigma). (B) Praf2 RNAi reduces etoposide-induced apoptosis also in MDA-MB 231cells. Cells were transfected with control or Praf2 targeting siRNAs. After 48 hours cells were treated for 24 hours with 50 µM etoposide. Cellular caspase 3 and 7 activities were measured using the Caspase-Glo 3/7 luminometric assay. The graph show the percentage of caspase 3 and 7 activation in samples treated with the Praf2 targeting siRNAs compared with the activity present in cells transfected with the control siRNA in 2 independent experiments. (TIF) [file pone.0015636.s002.tif]

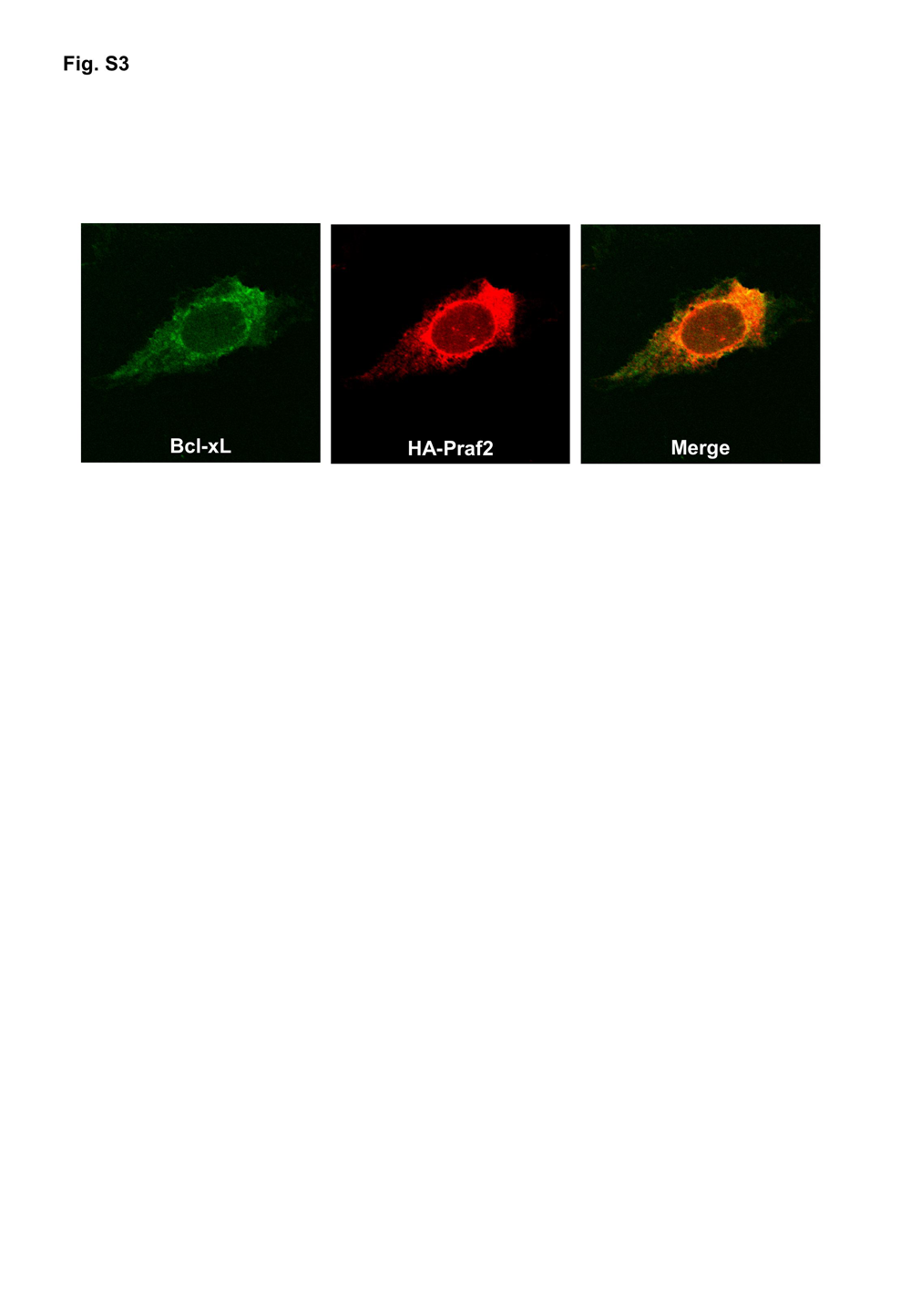

Supplement: Figure S3 — Praf2 partially co-localises with Bcl-xL in U2OS cells. U2OS cells were transfected with HA-Praf2 and analysed by confocal immunofluorescence microscopy using an antibody directed against Bcl-xL (Pharmingen) and an antibody recognising the HA-tag (Sigma). (TIF) [file pone.0015636.s003.tif]
